# Inhibition of HDAC8 mitigates AKI by reducing DNA damage and promoting homologous recombination repair

**DOI:** 10.1111/jcmm.70114

**Published:** 2024-09-24

**Authors:** Yanjin Wang, Chao Yu, Jianjun Yu, Fengchen Shen, Xinyu Du, Na Liu, Shougang Zhuang

**Affiliations:** ^1^ Department of Nephrology, Shanghai East Hospital, School of Medicine Tongji University Shanghai China; ^2^ Department of Medicine, Rhode Island Hospital and Alpert Medical School Brown University Providence Rhode Island USA

**Keywords:** acute kidney injury, apoptosis, cisplatin, DNA damage, HDAC8, homologous recombination repair

## Abstract

Nephrotoxicity is a major side effect of platinum‐based antineoplastic drugs, and there is currently no available therapeutic intervention. Our study suggests that targeting histone deacetylase 8 could be a potential treatment for cisplatin‐induced acute kidney injury (AKI). In a murine model of AKI induced by cisplatin, the administration of PCI‐34051, a selective inhibitor of HDAC8, resulted in significant improvement in renal function and reduction in renal tubular damage and apoptosis. Pharmacological inhibition of HDAC8 also decreased caspase‐3 and PARP1 cleavage, attenuated Bax expression and preserved Bcl‐2 levels in the injured kidney. In cultured murine renal epithelial cells (mRTECs) exposed to cisplatin, treatment with PCI‐34051 or transfection with HDAC8 siRNA reduced apoptotic cell numbers and diminished expression of cleaved caspase‐3 and PARP1; conversely, overexpression of HDAC8 intensified these changes. Additionally, PCI‐34051 reduced p53 expression levels along with those for p21, p‐CDK2 and γ‐H2AX while preserving MRE11 expression in the injured kidney. Similarly, pharmacological and genetic inhibition of HDAC8 reduced γ‐H2AX and enhanced MRE11 expression; conversely, HDAC8 overexpression exacerbated these changes in mRTECs exposed to cisplatin. These results support that HDAC8 inhibition attenuates cisplatin‐induced AKI through a mechanism associated with reducing DNA damage and promoting its repair.

## 1 INTRODUCTION

Cisplatin is a common chemotherapy drug for cancer, but it can cause serious kidney side effects, leading to AKI and chronic kidney disease (CKD).^1^ The exact mechanism of cisplatin‐induced nephrotoxicity is not fully understood, but it involves DNA damage, mitochondrial issues, oxidative stress, endoplasmic reticulum stress, autophagy, cell‐cycle arrest, apoptosis and inflammation.^1^ Recent research has also highlighted the role of epigenetics in AKI pathogenesis, with various histone modifications such as methylation, acetylation, phosphorylation and ubiquitination occurring in kidney cells during AKI.^2^, ^3^, ^4^ Thus, understanding the epigenetic mechanisms in AKI may offer new treatment strategies.

Histone acetylation is a crucial post‐translational modification that is reciprocally regulated by histone acetyltransferases (HATs) and histone deacetylases (HDACs). HATs facilitate the transfer of acetyl groups to histone lysine residues, a process typically associated with transcriptional activation, while histone deacetylases are responsible for removing acetyl groups, which normally inhibit gene expression.^5^, ^6^ To date, 18 mammalian HDACs have been identified. Based on the conservation of deacetylase domains and their dependence on specific cofactors, this family can be divided into four classes: class I (HDAC1, 2, 3 and 8), class II (HDAC4, 5, 6, 7, 9 and 10) and class IV (HDAC11), which are zinc‐dependent amide hydrolases; and class III (sirtuin1‐7), which require nicotinamide adenine dinucleotide (NAD^+^) as a cofactor for their catalytic function.^5^ The activation of HDAC is involved in multiple biological processes such as cell division, differentiation, proliferation, migration, epithelial–mesenchymal transition (EMT) and apoptosis.^7^, ^8^, ^9^ Although HDACs share some functions in some cases, each of classes and forms may have different functions.

Functional studies have revealed that HDAC8 plays a pivotal role in the pathogenesis of various cancers, but its involvement in other diseases is less well understood. Recent reports have indicated the potential involvement of HDAC8 in renal fibrosis. For instance, Choi et al. found that siRNA‐mediated silencing of HDAC8 reduces the expression of E‐cadherin in renal tubular epithelial cells, which is a critical molecular basis for EMT.^10^ Long et al. demonstrated that PCI‐34051, a selective inhibitor of HDAC8, reduced collagen production and inhibited the progression of fibrosis induced by nephrectomy in mice.^11^ Additionally, our recent study showed upregulation of HDAC8 in the kidneys of a murine model with renal fibrosis induced by unilateral ureteral obstruction (UUO), and administration of PCI‐34051 attenuated renal fibrosis.^12^ These findings suggest that HDAC8 is critically involved in the pathogenesis of chronic kidney diseases. However, the role of HDAC8 in AKI is not well elucidated and results from several in vitro studies are controversial. An early study demonstrated that overexpression of wild‐type HDAC8, but not a deacetylase‐defective mutant, attenuates tubular cell damage induced by hypoxia/reoxygenation and cobalt chloride in human proximal tubular HK‐2 cells.^13^ A recent study showed that treatment with PCI‐34051 reduces cisplatin‐induced production of inflammatory cytokines in immortalized human proximal tubule cells, suggesting a detrimental role for HDAC8.^11^ Given the contradictory results from studies on renal epithelial cells, further research is necessary to clarify the role of HDAC8 in both in vitro and in vivo models of AKI.

In this study, we utilized a murine model of cisplatin‐induced AKI to investigate the role and mechanism of HDAC8 in cisplatin nephrotoxicity. Our results demonstrate that HDAC8 is critically involved in the pathogenesis of AKI and renal epithelial cell apoptosis through a mechanism associated with DNA damage and maladaptive repair.

## 2 MATERIALS AND METHODS

### 2.1 Antibodies and reagents

PCI‐34051 was procured from Selleck (S2012, Selleck, Houston, USA). Fetal bovine serum was obtained from Biological Industries (Kibbutz Beth Haemek, Israel). Dulbecco's modified Eagle's medium (DMEM) and DMEM/F12 were purchased from Thermo Fisher Scientific (Waltham, MA, USA). Creatinine and urea assay kits were acquired from Nanjing Jiancheng Bioengineering Institute (Nanjing, China). The TUNEL assay kit was sourced from Beyotime (Shanghai, China), while the CCK8 solution was obtained from Dojindo Laboratories (Kumamoto, Japan). HDAC8 siRNA and scramble siRNA were both purchased from GenePharma (Shanghai, China). The following primary antibodies were utilized: HDAC8 (17548‐1‐AP; Proteintech), anti‐acetyl lysine rabbit mAb (PTM‐105RM; PTM BIO), γ‐H2AX (ab81299; Abcam), NGAL (sc‐515,876; Santa Cruz), cleaved PARP1 (94885S; Cell Signaling Technology), cleaved caspase‐3 (9664S; Cell Signaling Technology), Bax (A19684; ABclonal), Bcl‐2 (26593‐1‐AP; Proteintech), histone H3 (GB11102; Servicebio), p53 (32532S; Cell Signaling Technology), p21 (64016S; Cell Signaling Technology), phospho (Thr160)‐CDK2 (2561S; Cell Signaling Technology), CDK2 (10122‐1‐AP; Proteintech), MRE11 (A4222; ABclonal), β‐actin (81115‐1‐AP; Proteintech) and α‐Tubulin (11224‐1‐AP; Proteintech).

### 2.2 Cell culture and treatment

Murine renal tubular epithelial cells (mRTECs) were cultured in DMEM/F12 medium supplemented with 5% fetal bovine serum (FBS), 100 U/mL penicillin and 100 μg/mL streptomycin in a 5% CO_2_ environment at 37°C. To investigate the impact of HDAC8 inhibition on cisplatin‐induced mRTEC cell injury, mRTEC cells were serum‐starved for 24 h using serum‐free DMEM/F12, followed by treatment with PCI‐34051 (5 μM) or DMSO in the presence or absence of cisplatin (20 μg/mL) for 24 h.

### 2.3 Animals and treatment

Male C57BL/6J mice (20–23 g) were obtained from Shanghai JieSiJie Laboratory Animal Co. Ltd. (Shanghai, China). A mouse model of cisplatin‐induced AKI was established by intraperitoneal injection of a single dose of cisplatin (25 mg/kg) in saline. The effect of HDAC8 inhibitor PCI‐34051 on AKI was investigated by administering PCI‐34051 (20 mg/kg in 5% DMSO, 40% PEG300, 5% Tween 80, 50% ddH_2_O) intraperitoneally before cisplatin injection and then daily thereafter. Mice were euthanized at 72 h after cisplatin injection, and kidney samples were collected for histological examination and western blot analyses. Serum samples were collected for the detection of creatinine and urea nitrogen levels in mice. All experimental procedures were approved by the Institutional Animal Care and Use Committee of Shanghai East Hospital, Tongji University, China.

### 2.4 Renal function analysis

The levels of creatinine and urea nitrogen were determined using creatinine and blood urea nitrogen (BUN) kits, respectively, in accordance with the manufacturer's instructions.

### 2.5 Assessment of tubular injury

The severity of injury was assessed using the following scoring system, which ranged from 0 to 4: 0: indicating a normal condition; 1: representing <25% of the area injured; 2: indicating an injury affecting 25%–50% of the area; 3: representing an injury affecting 50%–75% of the area; 4: indicating an injury affecting >75% of the area.

### 2.6 Assessment of apoptotic cell

To assess apoptotic cell death, a TUNEL staining kit from Beyotime Biotechnology was utilized in accordance with the provided instructions. The quantification of TUNEL‐positive cells per field was conducted.

### 2.7 Cell counting kit‐8 (CCK‐8) assay

Cell viability was assessed using the CCK‐8 assay (Dojindo, Japan) following the manufacturer's instructions. Cells were plated in 96‐well plates with specific treatments and then incubated with CCK‐8 solution for 2 h. The absorbance at 450 nm (OD450) was measured using a microplate reader.

### 2.8 Western blot analysis

Kidney tissue samples and cultured cells were lysed using RIPA with a protease inhibitor cocktail. Following quantification of the protein concentration, 20–50 μg of protein was loaded and separated by SDS–PAGE gel electrophoresis, and then transferred to a PVDF membrane. The membrane was blocked with 5% nonfat milk for 1 h at room temperature, followed by incubation with specific primary antibodies at 4°C overnight. After washing with TBST, the membrane was incubated with HRP‐linked secondary antibody for 1 h at room temperature. Subsequently, the membranes were developed using enhanced chemiluminescence (ECL) western blot detection reagents. The chemiluminescent signals were visualized using the Tanon 4600 chemiluminescence imaging system. Band intensity was analysed using ImageJ software.

### 2.9 Histochemical and immunofluorescence

Staining kidney tissues were fixed in 4% paraformaldehyde for over 24 h, dehydrated in an ethanol series, cleared with xylene and subsequently embedded in paraffin. The paraffin‐embedded tissues were cut into 4 μm thickness for periodic acid‐Schiff (PAS) staining. For tissue immunofluorescent staining, the indicated primary antibodies were incubated overnight at 4°C followed by incubation with fluorescent‐conjugated secondary antibodies for 1 h at room temperature. Slides were viewed with a Leica DM6000B microscope.

### 2.10 Cell transfection

When the cells reached 50%–60% density, HDAC8 siRNA (Genepharma Inc., Shanghai, China) was transfected into the cells using Lipofectamine 2000 (Invitrogen‐Thermo Fisher Scientific, Carlsbad, CA, USA) according to the manufacturer's protocol. After 8 h, FBS‐free medium was changed to DMEM/F12 containing 5% FBS with penicillin and streptomycin. The knockdown cells were used for subsequent cisplatin treatment.

### 2.11 Establishment of stable cell line expressing HDAC8

The mouse coding sequence of HDAC8 was cloned into the plvx vector. HDAC8 lentivirus plasmids were packaged in HEK‐293 T cells by co‐transfection with the packaging plasmids psPAX2 and pMD2.G. Three days later, the supernatant was collected and used to infect mRTEC cells with 8 μg/mL polybrene. Stable cells were selected with medium supplemented with 5 μg/mL puromycin for 7 days and then used for subsequent experiments.

### 2.12 Statistical analysis

Data in this study are represented as mean ± S.E.M. The differences between two groups were compared by Student's *t*‐test. A *p*‐value of less than 0.05 was considered statistically significant difference between mean values.

## 3 RESULTS

### 3.1 Pharmacological inhibition of HDAC8 alleviates cisplatin‐induced AKI

**FIGURE 1:**
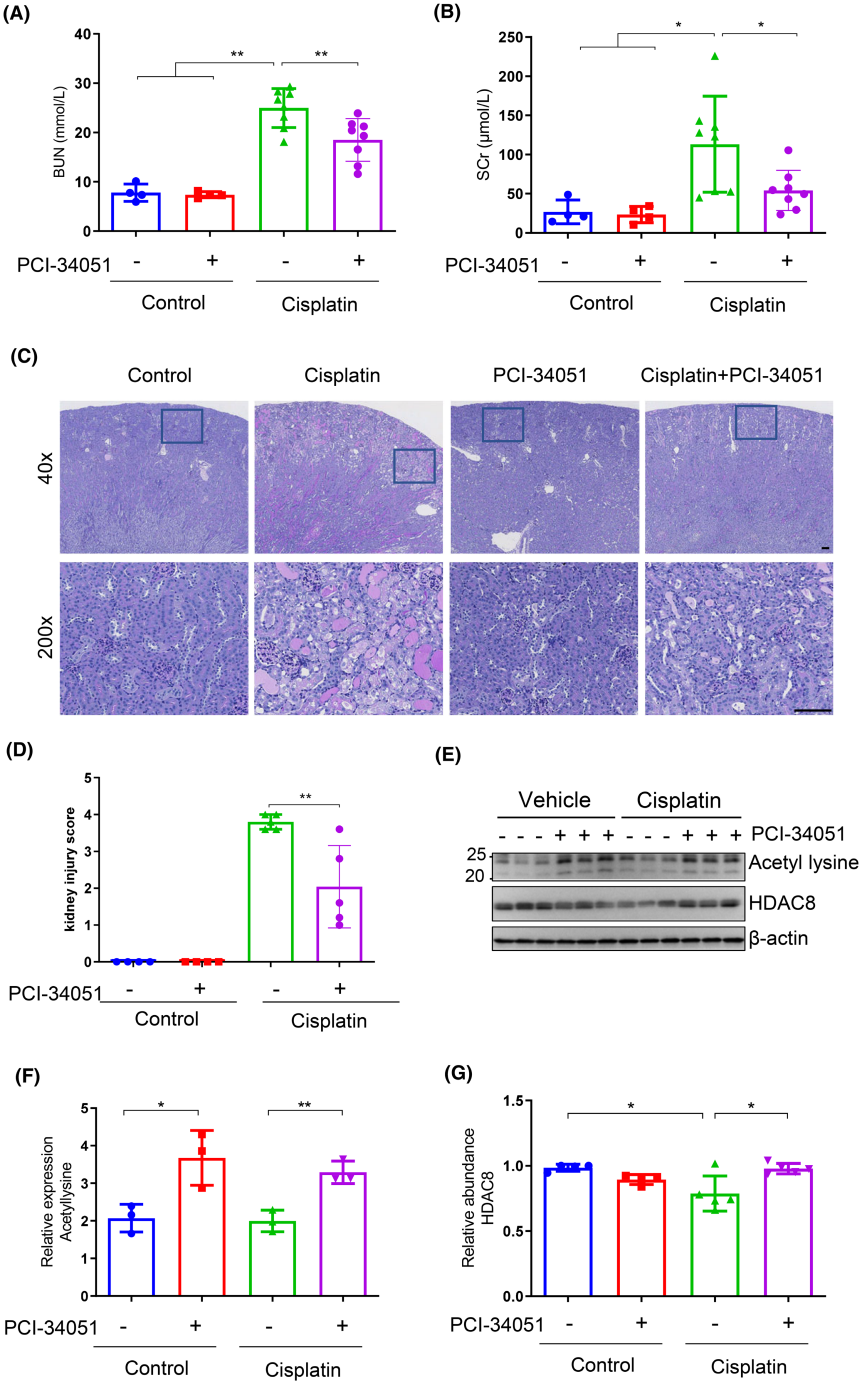
Inhibition of HDAC8 attenuates cisplatin‐induced AKI (A) Blood urea nitrogen (BUN) levels were measured using a colorimetric method in each group of mice. (B) Serum creatinine levels were detected by colorimetric method in each group of mice. (C) Periodic acid‐Schiff (PAS) staining revealed kidney structure damage in mice from each group. Scale bar = 100 μm. (D) Tubule injury scores of renal tissue were assessed in each group. (E) Western blot analysis was performed to detect the expression of lysine acetylated proteins and HDAC8 in kidney tissues from mice in each group. (F) The relative abundance of lysine acetylated proteins in immunoblots was quantified by densitometric ratios of lysine acetylated proteins/Tubulin. (G) The relative abundance of HDAC8 in immunoblots was quantified by densitometric ratios of HDAC8/Tubulin. Data are presented as mean ± standard deviations. *p < 0.05, **p < 0.01.

To assess the role of HDAC8 in cisplatin‐induced AKI, a model of cisplatin‐induced AKI was established in mice. The mice were intraperitoneally injected with cisplatin (25 mg/kg) for 3 days. A highly selective HDAC8 inhibitor, PCI‐34051, was utilized to inhibit HDAC8 activity. At 72 h after cisplatin injection, the mice were euthanized and serum and kidneys were collected for examination of serum creatinine, BUN and renal histopathology. Compared with the control group, administration of cisplatin led to an increase in the levels of serum creatinine and BUN (Figure 1A,B). However, treatment with PCI‐34051 resulted in decreased levels of serum creatinine and BUN following cisplatin injection (Figure 1A,B). Furthermore, PCI‐34051 reduced renal tubular damage as indicated by PAS staining; the tubular injury score demonstrated less tubular damage in kidneys exposed to both cisplatin and PCI‐34051 compared to those treated with cisplatin alone (Figure 1C,D). Injection of PCI‐34051 alone did not affect renal function or pathological damage. To evaluate the efficacy of PCI‐34051 in vivo, an anti‐acetyl lysine antibody was used to detect acetylated protein levels in kidney tissue. The results showed that PCI‐34051 increased acetylation levels in kidney tissue, indicating its effectiveness (Figure 1E,F). It is noteworthy that renal expression of HDAC8 declined after administration of cisplatin (Figure 1E–G). Immunohistochemistry staining revealed that HDAC8 was predominantly expressed in the nucleus, with some presence also detected in the cytoplasm (Figure **S1**). Immunostaining of cultured mRTECs also demonstrated a similar cellular distribution of HDAC8 (Figure S2). In summary, these findings suggest that activation of HDAC8 is involved in the pathogenesis of cisplatin‐induced AKI and that pharmacological inhibition of HDAC8 is renoprotective.

### 3.2 Pharmacological inhibition of HDAC8 reduces renal tubular injury in the kidneys following cisplatin treatment

**FIGURE 2:**
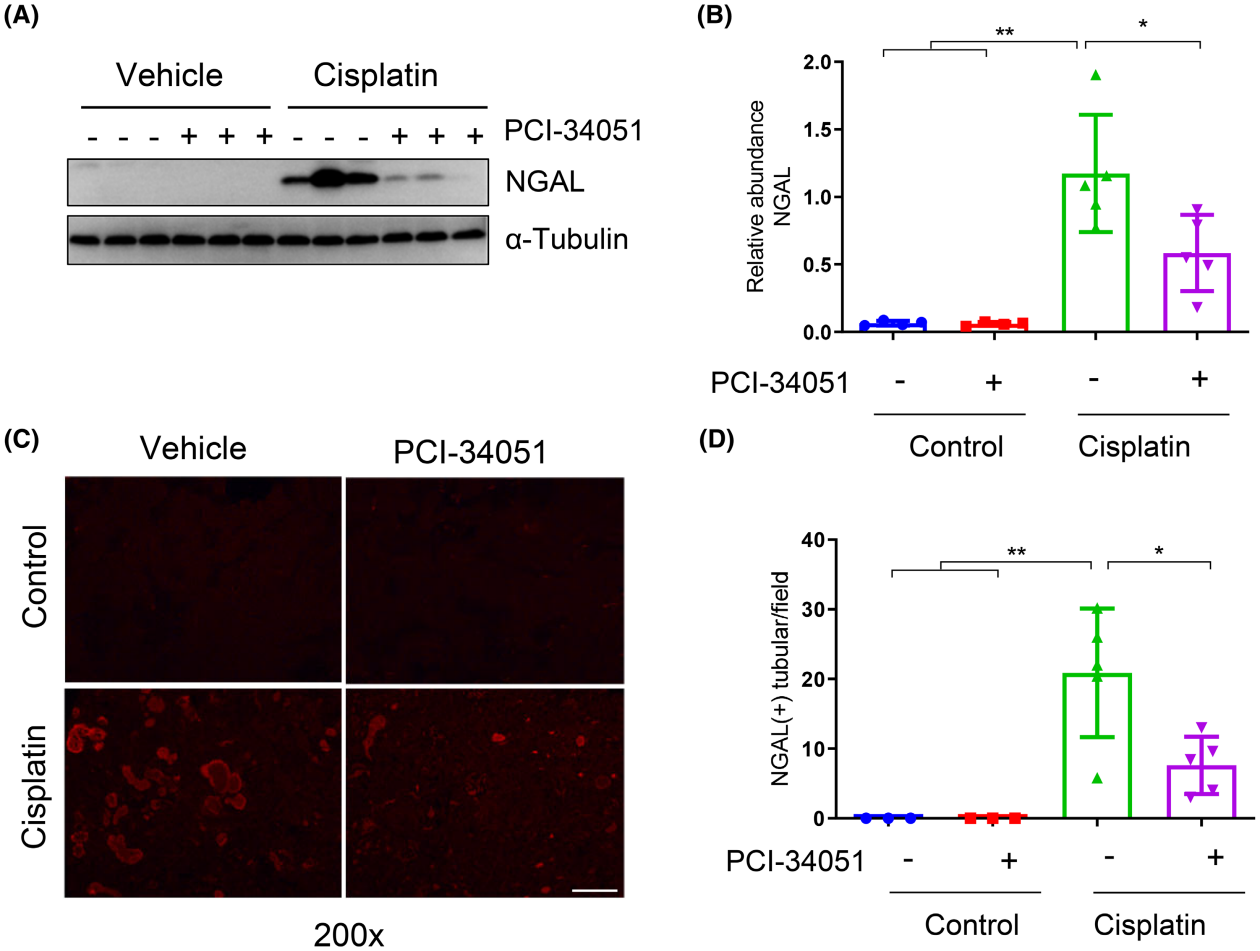
HDAC8 inhibition reduces NGAL expression levels. (A) Western blot analysis was performed to detect the expression of NGAL in kidney tissues of mice from each group. (B) Relative abundance of NGAL protein expression levels compared to Tubulin. (C) Immunofluorescence staining revealed the expression of NGAL in kidney tissue from each group. Scale bar = 100 μm. (D) Comparison of the number of cells positive for NGAL immunofluorescence staining in each group. Data are presented as mean ± standard deviations. *p < 0.05, **p < 0.01.

Neutrophil gelatinase‐associated lipocalin (NGAL) is recognized as an early biomarker of kidney injury, we thus examined its expression by immunoblot analysis and immunofluorescence staining. NGAL was not expressed in the murine kidneys without cisplatin treatment; but markedly increased following cisplatin administration. PCI‐34051 treatment largely suppressed its expression as shown by immunoblot analysis and confirmed by immunofluorescent staining (Figure 2A–D). These results suggest that HDAC8 activation contributes to renal tubular injury.

### 3.3 Pharmacological inhibition of HDAC8 reduces apoptosis in the kidneys following cisplatin treatment

**FIGURE 3:**
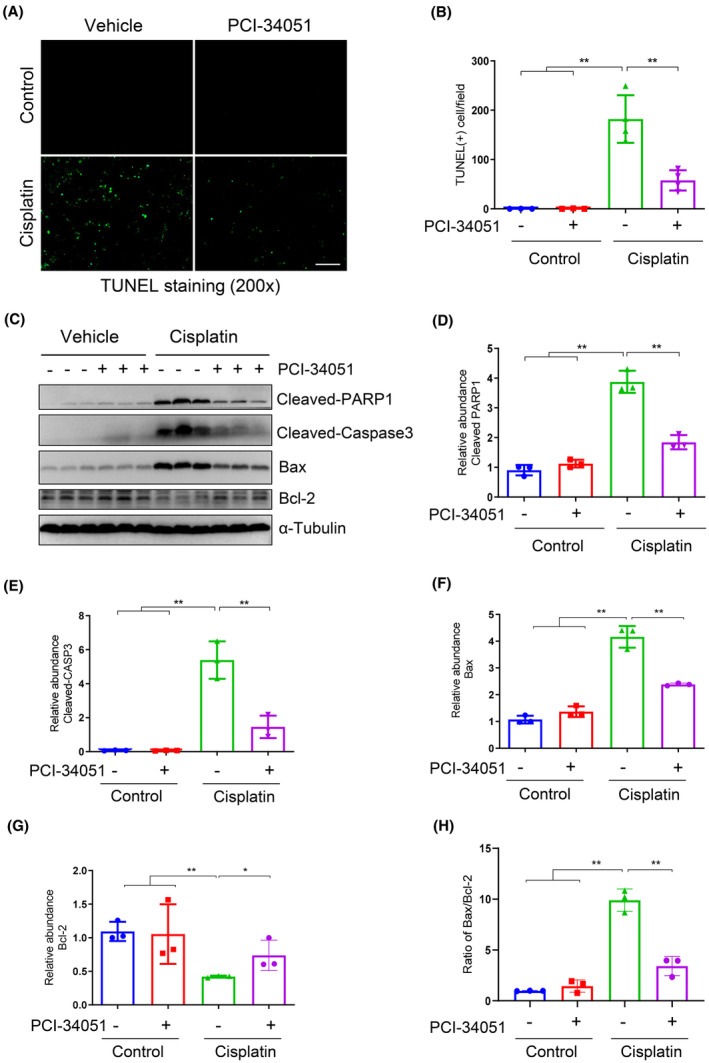
Inhibition of HDAC8 attenuates apoptosis of renal tubular epithelial cells. (A) TUNEL staining was performed on kidney tissue sections from mice in each group. Scale bar = 100 μm. (B) Statistical analysis showing the number of TUNEL‐staining positive cells in each group. (C) Western blot analysis was used to detect the expression of cleaved PARP1, cleaved caspase‐3, Bax and Bcl‐2 in the kidney tissues from mice in each group. (D–G) Relative abundance of cleaved PARP1, cleaved caspase‐3, Bax and Bcl‐2 protein expression levels compared to Tubulin. Data are expressed as mean ± standard deviations.*p < 0.05, **p < 0.01.

To investigate the role of HDAC8 in apoptosis in the kidneys induced by cisplatin, we examined the effect of HDAC8 inhibition on apoptosis of kidney cells using TUNEL staining and immunoblot analysis. Our findings revealed that compared to mice without PCI‐34051 treatment after cisplatin injection, the number of TUNEL‐positive renal tubular cells was significantly reduced in mice treated with PCI‐34051 (Figure 3A,B). No apoptotic cells were observed in the kidneys of control or PCI‐34051 alone treated mice (Figure 3A,B). Furthermore, western blot analysis demonstrated that PCI‐34051 treatment significantly reduced cisplatin‐induced cleavage of PARP1 and caspase‐3, two major proteins associated with apoptosis (Figure 3C–E). Given that the mitochondrial apoptotic pathway plays a crucial role in nephrotoxic AKI,^14^ we also examined the expression levels of Bax and Bcl‐2 in the kidney. Bax plays a pivotal role in mitochondrial membrane permeabilization, while Bcl‐2 is an anti‐apoptotic survival molecule expressed in mitochondria. As depicted in Figure 3C,F,G, the expression level of Bax increased while that of Bcl‐2 decreased following cisplatin treatment. Conversely, PCI‐34051 exhibited a significant reduction in Bax expression and an increase in Bcl‐2 expression. Furthermore, PCI‐34051 also reduced the ratio of Bax/Bcl‐2 (Figure 3H). These results indicate that inhibition of HDAC8 protects against apoptosis in the kidney following exposure to cisplatin.

### 3.4 Pharmacological inhibition of HDAC8 reduces renal tubular cell apoptosis and improves cell viability in culture

**FIGURE 4:**
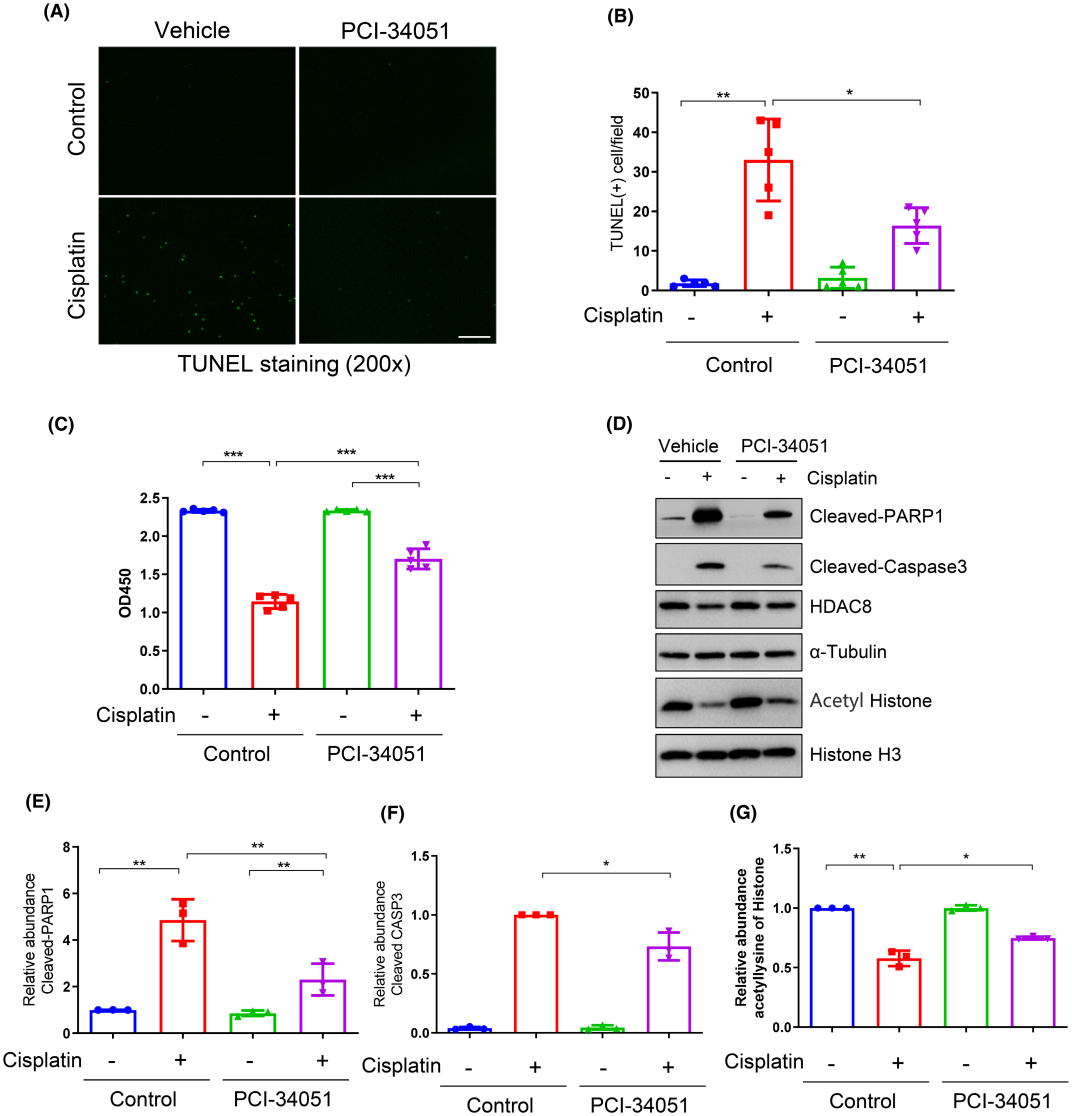
HDAC8 blockade alleviates renal tubular epithelial cell apoptosis and increases cell viability. (A) TUNEL staining of renal tubular epithelial cells in each group. Scale bar = 100 μm. (B) Comparison of the number of TUNEL‐staining positive cells in each group. (C) CCK8 was used to detect cell viability in each group. (D) Western blotting to detect the expression of cleaved PARP1, cleaved caspase‐3, and lysine acetylated histone of renal tubular epithelial cell in each group. (E) Relative abundance of cleaved PARP1 protein expression levels. (F) Relative abundance of cleaved caspase‐3 protein expression levels. (G) Relative abundance of lysine acetylated histone protein. Data are expressed as mean ± standard deviations. *p < 0.05, **p < 0.01, ***p < 0.001.

To validate the impact of HDAC8 inhibition on the apoptosis of renal tubular cells, we investigated the effect of PCI‐34051 on apoptosis and cell viability of murine renal tubular epithelial cells (mRTEC) following cisplatin exposure in vitro. The results revealed that TUNEL‐positive mRTECs were present in cisplatin‐exposed cells; however, treatment with PCI‐34051 significantly reduced this population of cells. No apoptotic cells were observed in the mRTECs treated with PCI‐34051 alone (Figure 4A,B). Furthermore, PCI‐34051 effectively promoted cell survival in cells exposed to cisplatin (Figure 4C). Consistent with our findings in vivo, PCI‐34051 also markedly inhibited cisplatin‐induced cleavage of PARP1 and caspase‐3 (Figure 4D–F). Additionally, the HDAC8 inhibitor increased the acetylation levels of histones compared to the group exposed to cisplatin without PCI‐34051 (Figure 4G). These results indicate that HDAC8 inhibition is capable of reducing apoptosis at the cellular level.

### 3.5 Effects of HDAC8 silencing and HDAC8 overexpression on cisplatin‐induced apoptosis in renal tubular epithelial cells

**FIGURE 5:**
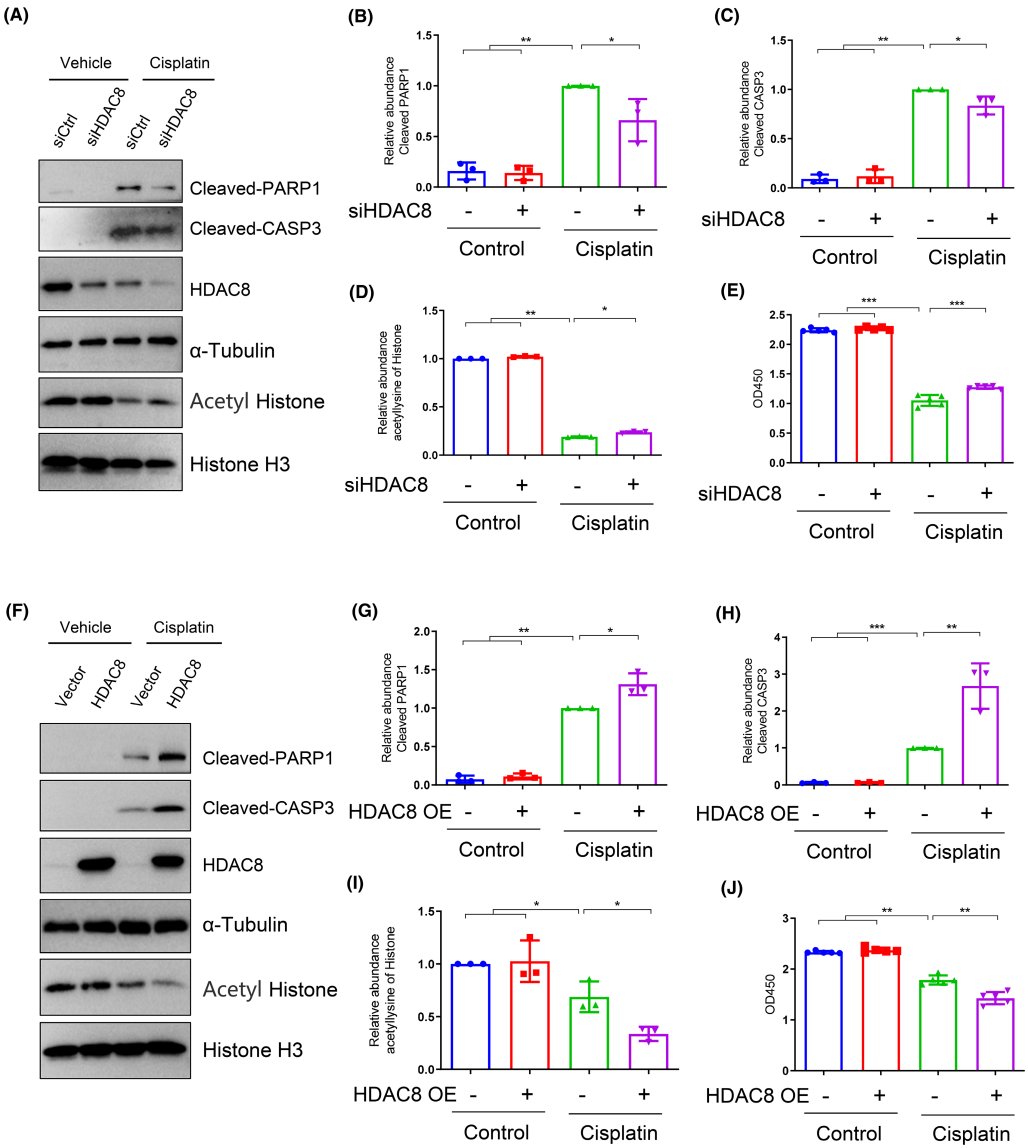
HDAC8 exacerbates histone deacetylation and promotes cisplatin‐induced apoptosis in renal tubular epithelial cells. (A) The protein levels of cleaved PARP1, cleaved caspase‐3, and acetylated histone protein in renal tubular epithelial cells in each group. Cells were treated with HDAC8 siRNA and control siRNA for 48 h, and then cultured in cisplatin‐containing medium for 24 h. (B) Relative abundance of cleaved PARP1 protein expression levels. (C) Relative abundance of cleaved caspase‐3 protein expression levels. (D) Relative abundance of lysine acetylated histone protein. (E) CCK8 was used to detect cell viability in each group. (F) Western blotting to detect the expression changes of the corresponding proteins of HDAC8 overexpression and control renal tubular epithelial cells in each group. (G) Relative abundance of cleaved PARP1 protein expression levels. (H) Relative abundance of cleaved caspase‐3 protein expression levels. (I) Relative abundance of lysine acetylated histone. (J): CCK8 was used to detect cell viability in each group. Data are expressed as mean ± standard deviations. *p < 0.05, **p < 0.01, ***p < 0.001.

To confirm the role of HDAC8 in cisplatin‐induced renal epithelial cell apoptosis, siRNA and HDAC8‐overexpressed mRTECs were utilized. Compared with cells transfected with scrambled siRNA, those transfected with HDAC8 siRNA exhibited reduced apoptosis and expression levels of cleaved PARP1 and cleaved caspase‐3 following exposure to cisplatin (Figure 5A–C). Moreover, HDAC8 knockdown increased histone acetylation levels upon exposure to cisplatin (Figure 5A,D). The viability of cells transfected with HDAC8 siRNA was higher than that observed in those transfected with scramble siRNA following exposure to cisplatin (Figure 5E). Conversely, HDAC8 overexpression decreased histone acetylation levels (Figure 5I) and exacerbated cisplatin‐induced apoptosis, as evidenced by increased levels of cleaved PARP1 and cleaved caspase‐3 in cells, and reduced cell viability (Figure 5F–H,J). These results further support the idea that HDAC8 is involved in the regulation of renal epithelial cell apoptosis.

### 3.6 Pharmacological inhibition of HDAC8 suppresses DNA damage response in the kidneys following cisplatin treatment

**FIGURE 6:**
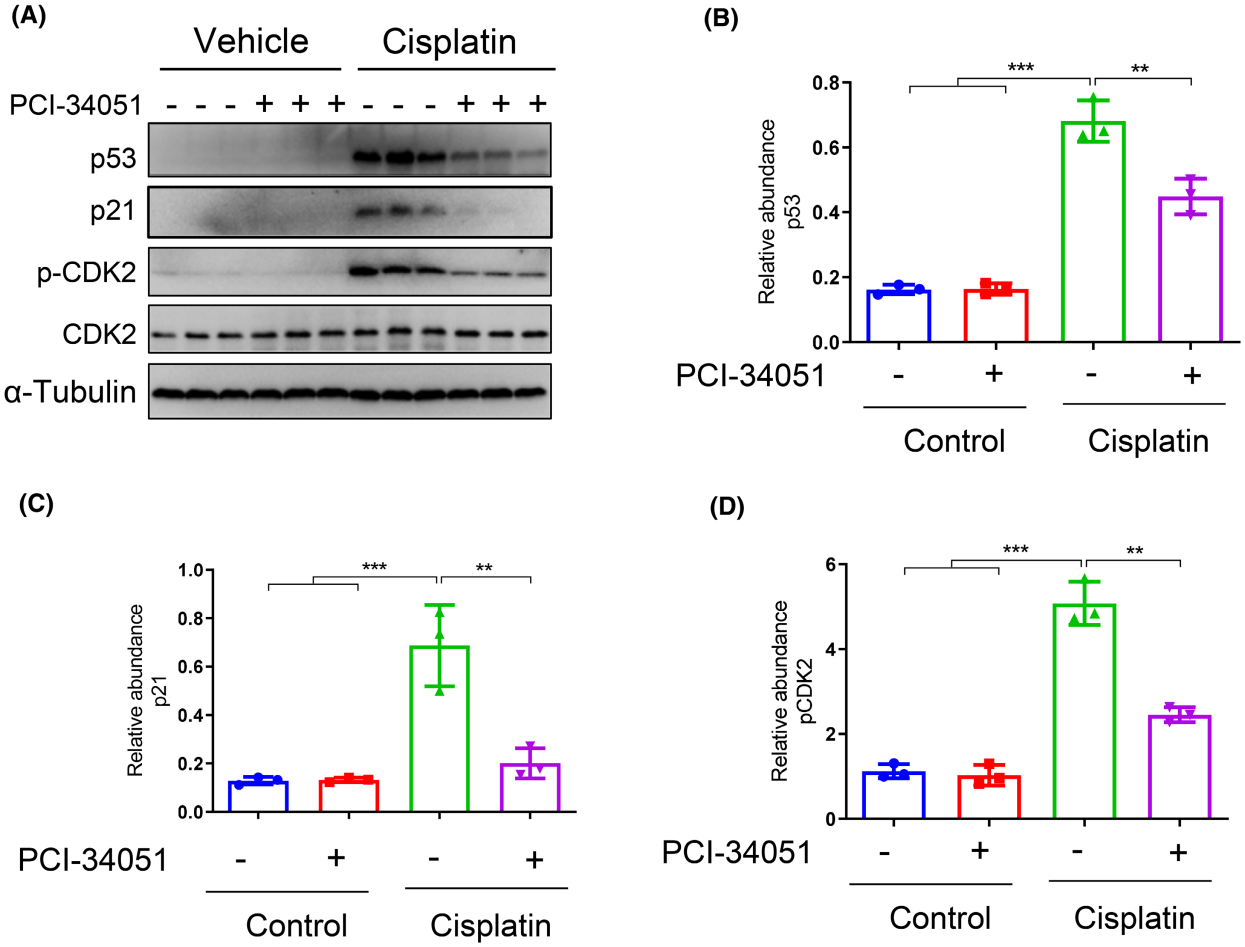
HDAC8 inhibition induces changes in the p53 signalling pathway. (A) The protein levels of p53, p21, p‐CDK2 and CDK2 in kidney tissue from each group are shown. (B) Relative abundance of p53 protein expression levels. (C) Relative abundance of p21 protein expression levels. (D) Relative abundance of p‐CDK2 protein expression levels. Data are presented as mean ± standard deviations. *p < 0.05, **p < 0.01.

DNA damage plays a crucial role in cisplatin‐induced AKI.^1^, ^15^, ^16^ The uptake of cisplatin in renal tubular cells causes DNA damage, ultimately leading to cell cycle arrest and apoptosis.^1^, ^15^, ^16^ Given that increased expression of p53 and p21, as well as phosphorylation of CDK2, is essential in regulating apoptosis and cell cycle arrest,^17^ we investigated the impact of HDAC8 inhibition on their expression in the kidney following cisplatin administration. As depicted in Figure 6, the expression of p53 and p21 was significantly elevated after cisplatin stimulation; however, PCI‐34051 inhibited their expression. Similarly, PCI‐34051 effectively suppressed cisplatin‐induced CDK2 phosphorylation. Since p53 can transcriptionally induce the expression of apoptotic genes such as Bax leading to caspase‐3 activation and cell death while increasing the expression of p21 resulting in senescence/cell cycle arrest,^18^ these results suggest that HDAC8 may transduce the DNA signal to associated kinases which subsequently regulate p53 activation and p21 expression leading to apoptosis and cell cycle arrest.

### 3.7 Inhibition of HDAC8 mitigates cisplatin‐induced DNA damage and enhances homologous recombination repair

**FIGURE 7:**
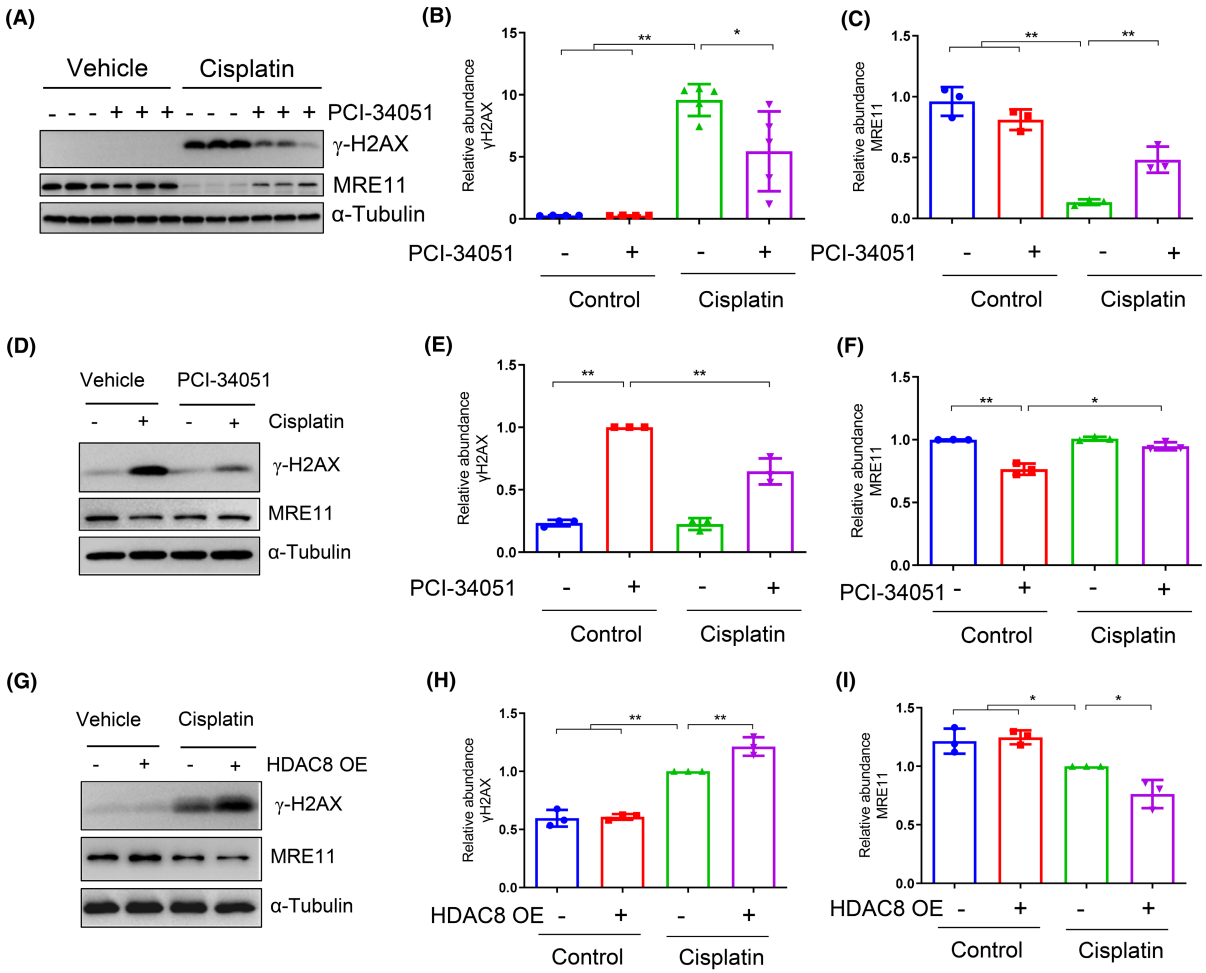
HDAC8 inhibition enhances homologous recombination repair. (A) Western blot analysis of γ‐H2AX protein levels in kidney tissues from each group. (B) Statistical analysis of relative abundance of γ‐H2AX expression levels. (C) Western blot analysis of MRE11 protein levels in kidney tissues from each group. (D) Statistical analysis of relative abundance of MRE11 expression levels. (E) Western blot analysis of γ‐H2AX and MRE11 protein levels in renal tubular epithelial cells from each group. (F) Statistical analysis of relative abundance of γ‐H2AX expression levels. (G) Statistical analysis of relative abundance of MRE11 expression levels. (H) Protein levels of γ‐H2AX and MRE11 in HDAC8 overexpressing renal tubular epithelial cells from each group. (I) Statistical analysis of relative abundance of γ‐H2AX expression levels. (J) Statistical analysis on the relative abundanceof MRE11expressionlevels. Data are presented as mean ± standard deviation. *p < 0.05, **p < 0.01.

Cisplatin not only disrupts DNA function by creating monoadducts and DNA crosslinks to eliminate cancer cells, but it also causes damage to normal tissue cells, particularly kidney tissue.^19^, ^20^ Therefore, we examined the molecular markers associated with DNA damage and repair. Following cisplatin stimulation, the expression of γ‐H2AX, an early marker for DNA double‐strand breaks (DSB), was increased, while MRE11, a protein associated with DNA homologous recombination (HR) repair,^21^ was reduced (Figure 7A–C). Administration of PCI‐34051 reduced the expression of γ‐H2AX, but partially resumed the expression of MRE11 in the kidney with cisplatin treatment (Figure 7A–C). Similarly, PCI‐34051 reduced the increased expression of γ‐H2AX and preserved MRE11 expression in cultured mRTEC exposed to cisplatin (Figure 7D–F). In contrast, overexpression of HDAC8 resulted in increased expression of γ‐H2AX and decreased MRE11 expression after exposure to cisplatin (Figure 7G–I). These results indicate that inhibition of HDAC8 alleviates DNA damage and promotes its repair in the kidney and cultured renal epithelial cells following cisplatin treatment.

## 4 DISCUSSION

Although our recent studies have demonstrated that HDAC8 mediates renal fibrosis, its role in AKI remains unclear. In this study, we revealed that HDAC8 activation contributes to AKI as evidenced by the following observations: (1) inhibition of HDAC8 with PCI‐34051 alleviated cisplatin‐induced tubular cell injury and apoptosis in mice; (2) PCI‐34051 was effective in inhibiting apoptosis in cultured renal epithelial cells exposed to cisplatin; and (3) siRNA‐mediated silencing of HDAC8 suppressed while HDAC8 overexpression enhanced cisplatin‐induced apoptosis of renal epithelial cells in culture. These results provide strong evidence that HDAC8 is a critical mediator in cisplatin‐induced AKI and suggest that HDAC8 is a potential therapeutic target for AKI.

HDAC8 is widely expressed in various tissues and cell lines.^22^ Unlike other isoforms of class I HDACs, HDAC8 is primarily located in the nucleus but also present in the cytoplasm.^23^, ^24^, ^25^, ^26^ Our previous studies have also demonstrated that among class I HDACs, HDAC1, 2 and 3 are expressed in the nuclei, while HDAC8 is found in both the nucleus and cytoplasm of normally cultured renal epithelial cells.^27^ In a murine model of renal fibrosis induced by UUO, HDAC8 was also detected in both cellular components of renal epithelial cells and its expression levels increased over time.^12^ We further examined HDAC8 expression in kidney cells injured by cisplatin. Although HDAC8 was observed in both the nucleus and cytoplasm of renal epithelial cells, its expression declined following cisplatin treatment. This suggests that HDAC8 expression varies with different insults. The mechanism responsible for reduced expression of HDAC8 in cisplatin‐treated kidneys and cells remains unclear; however, HDAC8 expression has been documented to be regulated at the level of transcription, translation or degradation.^28^ For instance, the transcription factor binds to the promoter of HDAC8 and activates its transcription,^29^ while autophagy and the ubiquitin‐proteasome system can induce HDAC8 degradation.^30^ Considering that cisplatin‐induced AKI is associated with repressed expression of YY1 and increased autophagy,^31^, ^32^ it would be intriguing to investigate whether the downregulation of HDAC8 in renal cells is regulated by these mechanisms in vivo and in vitro following cisplatin stimulation. It is possible that reducing HDAC8 expression after exposure to cisplatin may represent an adaptive response aimed at minimizing damage signal transduction to renal epithelial cells. In support of this hypothesis, we have demonstrated that inhibition of HDAC8 activity using PCI‐34051 attenuated cisplatin‐induced renal tubular injury and apoptosis both in vivo and in vitro.

Given its multiple cellular distribution, HDAC8 may catalyse the deacetylation of substrates located in both the nucleus and cytoplasm.^33^ Previous studies have reported that HDAC8 is capable of catalysing the deacetylation of H4K16ac and H4K20ac on histone H4,^24^, ^34^ as well as H3K14ac and H3K16ac on histone H3.^33^ Additionally, HDAC8 has been shown to induce deacetylation of certain non‐histone proteins such as SMC3, ERRα, cAMP response element‐binding protein (CREB) and p53. It is important to note that these histone and non‐histone proteins, with the exception of SMC3, are not only deacetylated by HDAC8 but also by other HDACs.^28^ Among the non‐histone substrates, HDAC8‐mediated deacetylation of p53 has been shown to promote the transformation of leukaemia stem cells and inhibits apoptosis.^35^ In our study, we observed that treatment of renal epithelial cells with HDAC8 siRNA and inhibitors inhibited histone deacetylation in renal tubular epithelial cells after cisplatin exposure in vitro (Figures 4 and 5) and reduced p53 expression in vivo (Figure 6). This suggests that HDAC8‐mediated deactylation is essential for maintaining p53 stability. Given that p53 plays a key role in apoptosis of renal epithelial cells following cisplatin exposure, its upregulation mediated by HDAC8 may contribute to the pathogenesis of AKI. Supporting this hypothesis, our data showed that cisplatin‐induced upregulation of p53 was accompanied by increased expression of p21 and apoptosis, which was largely suppressed by PCI‐34051 in injured kidneys. p21 acts as an inhibitor for cyclin‐dependent kinase thereby inhibiting cell cycle progression during G1 and S phases while triggering apoptosis.^36^

The activation of HDAC8 may contribute to apoptosis and renal injury through the regulation of DNA damage. Our study revealed that the expression of γ‐H2AX was elevated following cisplatin treatment, whereas administration of PCI‐34051 significantly reduced its expression (Figure 7). This suggests that HDAC8 activation is involved in the process of DNA damage response (DDR), as γ‐H2AX is an early step in DDR and essential for initiating the cellular response to DNA damage.^37^ DDR is a signalling pathway activated by DSBs, recruiting signalling proteins to chromatin to regulate DNA repair, replication stress responses and apoptosis. HR repair is a major mechanism for DSB repair in response to DNA damage,^33^ but its role in AKI has been rarely reported. While Kim et al. found that low doses of cisplatin increased expression of MRE11 and MRN complex molecules,^38^ our study demonstrated a reduction in MRE11 levels in the kidney and renal epithelial cells following high‐dose cisplatin treatment; these contrasting results may be attributed to differences in cisplatin dosage used between studies. Furthermore, HDAC8 inhibition partially resumed MRE11 expression. Given the crucial role of MRE11 in HR pathway initiation, it is possible that HDAC8 contributes to apoptosis through deficient repair mechanisms related to HR pathways. However, further research is needed to elucidate how exactly HDAC8 functions within this intricate process involving multiple molecules and events associated with HR repair.

Previous studies have heavily focused on the role of HDAC8 in cancer progression. There is increasing evidence indicating that HDAC8 is upregulated in cancer and associated with tumour cell proliferation, metastasis, immune evasion and drug resistance. Consequently, HDAC8 inhibitors have been tested alone or in combination with other inhibitors in various types of cancer. For instance, PCI‐34051 has been shown to induce caspase‐dependent apoptosis in T‐cell lymphomas and leukaemias,^39^ and its combination with ACY‐241 (a selective HDAC6 inhibitor) synergistically enhances apoptosis in ovarian cancer cells.^40^ Other HDAC8‐selective inhibitors such as 1,3,4‐oxadiazole‐alanine hybrid and BMX also induce cell apoptosis in breast cancer cells and colorectal cancer cells.^41^ Additionally, siRNA‐mediated depletion of HDAC8 has been found to increase the rate of apoptosis in gastric cancer cells.^42^ These findings suggest that contrary to the renoprotective effect of HDAC8 inhibition, pharmacological or genetic inhibition of HDAC8 exerts an anti‐cancer effect both in vitro and in vivo by inducing apoptosis of cancer cells. Given that cisplatin is widely used for chemotherapy across various tumours, the renoprotective effects of PCI‐34051 identified in this study suggest a promising possibility: targeting HDAC8 may enhance the anti‐tumour effects of cisplatin while simultaneously protecting the kidneys during cisplatin chemotherapy‐achieving the goal of ‘killing two birds with one stone.’

In summary, we are the first to demonstrate the role of HDAC8 in mediating cisplatin‐induced AKI and renal epithelial cell apoptosis. Mechanistically, HDAC8 may induce DNA damage response which subsequently upregulates p53 and p21‐leading to cell cycle arrest as well as renal epithelial cell apoptosis. Therefore, inhibiting HDAC8 may be a promising therapeutic approach for protecting kidneys while potentiating the anti‐tumour effects of cisplatin among tumour patients.

## AUTHOR CONTRIBUTIONS

**Yanjin Wang:** Data curation (equal); investigation (equal); methodology (equal); validation (equal); writing – original draft (equal). **Chao Yu:** Investigation (equal); methodology (equal). **Jianjun Yu:** Investigation (equal). **Fengchen Shen:** Investigation (equal). **Xinyu Du:** Investigation (equal). **Na Liu:** Supervision (equal). **Shougang Zhuang:** Conceptualization (equal); funding acquisition (equal); project administration (equal); resources (equal); supervision (equal); writing – review and editing (equal).

## FUNDING INFORMATION

This study was supported by the National Natural Science Foundation of China (82200818 to C.Y., and 82370698 and 82070700 to S.Z.).

## CONFLICT OF INTEREST STATEMENT

The authors declare no conflicts of interest.

## Supporting information


Data S1.

## Data Availability

The raw data supporting the conclusions of this article will be made available by the authors on request.

## References

[1] Tang C , Livingston MJ , Safirstein R , Dong Z . Cisplatin nephrotoxicity: new insights and therapeutic implications. Nat Rev Nephrol. 2023;19(1):53‐72. doi:10.1038/s41581-022-00631-7 36229672

[2] Wang J , Shen F , Liu F , Zhuang S . Histone modifications in acute kidney injury. Kidney Dis (Basel). 2022;8(6):466‐477. doi:10.1159/000527799 36590679 PMC9798838

[3] Zhou X , Chen H , Shi Y , Ma X , Zhuang S , Liu N . The role and mechanism of histone deacetylases in acute kidney injury. Front Pharmacol. 2021;12:695237. doi:10.3389/fphar.2021.695237 34220520 PMC8242167

[4] Tang J , Zhuang S . Epigenetics in acute kidney injury. Curr Opin Nephrol Hypertens. 2015;24(4):351‐358. doi:10.1097/MNH.0000000000000140 26050122 PMC4559861

[5] Ellmeier W , Seiser C . Histone deacetylase function in CD4(+) T cells. Nat Rev Immunol. 2018;18(10):617‐634. doi:10.1038/s41577-018-0037-z 30022149

[6] Haberland M , Montgomery RL , Olson EN . The many roles of histone deacetylases in development and physiology: implications for disease and therapy. Nat Rev Genet. 2009;10(1):32‐42. doi:10.1038/nrg2485 19065135 PMC3215088

[7] Johnstone RW . Histone‐deacetylase inhibitors: novel drugs for the treatment of cancer. Nat Rev Drug Discov. 2002;1(4):287‐299. doi:10.1038/nrd772 12120280

[8] Jamaladdin S , Kelly RD , O'Regan L , et al. Histone deacetylase (HDAC) 1 and 2 are essential for accurate cell division and the pluripotency of embryonic stem cells. Proc Natl Acad Sci USA. 2014;111(27):9840‐9845. doi:10.1073/pnas.1321330111 24958871 PMC4103379

[9] Li Y , Seto E . HDACs and HDAC inhibitors in cancer development and therapy. Cold Spring Harb Perspect Med. 2016;6(10):a026831. doi:10.1101/cshperspect.a026831 27599530 PMC5046688

[10] Choi SY , Kee HJ , Kurz T , et al. Class I HDACs specifically regulate E‐cadherin expression in human renal epithelial cells. J Cell Mol Med. 2016;20(12):2289‐2298. doi:10.1111/jcmm.12919 27420561 PMC5134402

[11] Long K , Vaughn Z , McDaniels MD , et al. Validation of HDAC8 inhibitors as drug discovery starting points to treat acute kidney injury. ACS Pharmacol Transl. 2022;5(4):207‐215. doi:10.1021/acsptsci.1c00243 PMC9003639 35434532

[12] Zhang YH , Zou JN , Tolbert E , Zhao TC , Bayliss G , Zhuang SG . Identification of histone deacetylase 8 as a novel therapeutic target for renal fibrosis. FASEB J. 2020;34(6):7295‐7310. doi:10.1096/fj.201903254R 32281211 PMC7445474

[13] Ha SD , Solomon O , Akbari M , Sener A , Kim SO . Histone deacetylase 8 protects human proximal tubular epithelial cells from hypoxia‐mimetic cobalt‐ and hypoxia/reoxygenation‐induced mitochondrial fission and cytotoxicity. Sci Rep‐UK. 2018;8(1):11332. doi:10.1038/s41598-018-29463-x PMC6063935 30054507

[14] Bock FJ , Tait SWG . Mitochondria as multifaceted regulators of cell death. Nat Rev Mol Cell Biol. 2020;21(2):85‐100. doi:10.1038/s41580-019-0173-8 31636403

[15] Zhu S , Pabla N , Tang C , He L , Dong Z . DNA damage response in cisplatin‐induced nephrotoxicity. Arch Toxicol. 2015;89(12):2197‐2205. doi:10.1007/s00204-015-1633-3 26564230 PMC4734632

[16] Yan M , Tang C , Ma Z , Huang S , Dong Z . DNA damage response in nephrotoxic and ischemic kidney injury. Toxicol Appl Pharmacol. 2016;313:104‐108. doi:10.1016/j.taap.2016.10.022 27984128 PMC5362306

[17] Engeland K . Cell cycle regulation: p53‐p21‐RB signaling. Cell Death Differ. 2022;29(5):946‐960. doi:10.1038/s41418-022-00988-z 35361964 PMC9090780

[18] Hodeify R , Tarcsafalvi A , Megyesi J , Safirstein RL , Price PM . Cdk2‐dependent phosphorylation of p21 regulates the role of Cdk2 in cisplatin cytotoxicity. Am J Physiol Renal Physiol. 2011;300(5):F1171‐F1179. doi:10.1152/ajprenal.00507.2010 21325496 PMC3094048

[19] Rottenberg S , Disler C , Perego P . The rediscovery of platinum‐based cancer therapy. Nat Rev Cancer. 2021;21(1):37‐50. doi:10.1038/s41568-020-00308-y 33128031

[20] Garaycoechea JI , Quinlan C , Luijsterburg MS . Pathological consequences of DNA damage in the kidney. Nat Rev Nephrol. 2023;19(4):229‐243. doi:10.1038/s41581-022-00671-z 36702905

[21] Groelly FJ , Fawkes M , Dagg RA , Blackford AN , Tarsounas M . Targeting DNA damage response pathways in cancer. Nat Rev Cancer. 2023;23(2):78‐94. doi:10.1038/s41568-022-00535-5 36471053

[22] Buggy JJ , Sideris ML , Mak P , Lorimer DD , McIntosh B , Clark JM . Cloning and characterization of a novel human histone deacetylase, HDAC8. Biochem J. 2000;350 Pt 1(Pt 1):199‐205. 10926844 PMC1221242

[23] Hu E , Chen Z , Fredrickson T , et al. Cloning and characterization of a novel human class I histone deacetylase that functions as a transcription repressor. J Biol Chem. 2000;275(20):15254‐15264. doi:10.1074/jbc.M908988199 10748112

[24] Van den Wyngaert I , de Vries W , Kremer A , et al. Cloning and characterization of human histone deacetylase 8. FEBS Lett. 2000;478(1–2):77‐83. doi:10.1016/s0014-5793(00)01813-5 10922473

[25] Li J , Chen S , Cleary RA , et al. Histone deacetylase 8 regulates cortactin deacetylation and contraction in smooth muscle tissues. Am J Physiol Cell Physiol. 2014;307(3):C288‐C295. doi:10.1152/ajpcell.00102.2014 24920679 PMC4121581

[26] Vanaja GR , Ramulu HG , Kalle AM . Overexpressed HDAC8 in cervical cancer cells shows functional redundancy of tubulin deacetylation with HDAC6. Cell Commun Signal. 2018;16(1):20. doi:10.1186/s12964-018-0231-4 29716651 PMC5930436

[27] Tang J , Yan Y , Zhao TC , Bayliss G , Yan H , Zhuang S . Class I histone deacetylase activity is required for proliferation of renal epithelial cells. Am J Physiol Renal Physiol. 2013;305(3):F244‐F254. doi:10.1152/ajprenal.00126.2013 23698124 PMC3742866

[28] Kim JY , Cho H , Yoo J , et al. Pathological role of HDAC8: cancer and beyond. Cells. 2022;11(19). doi:10.3390/cells11193161 PMC9563588 36231123

[29] Fu W , Liang D , Wu X , et al. Long noncoding RNA LINC01435 impedes diabetic wound healing by facilitating YY1‐mediated HDAC8 expression. iScience. 2022;25(4):104006. doi:10.1016/j.isci.2022.104006 35330681 PMC8938286

[30] Park JY , Juhnn YS . cAMP signaling increases histone deacetylase 8 expression by inhibiting JNK‐dependent degradation via autophagy and the proteasome system in H1299 lung cancer cells. Biochem Biophys Res Commun. 2016;470(2):336‐342. doi:10.1016/j.bbrc.2016.01.049 26792731

[31] Yang C , Xu H , Yang D , et al. A renal YY1‐KIM1‐DR5 axis regulates the progression of acute kidney injury. Nat Commun. 2023;14(1):4261. doi:10.1038/s41467-023-40036-z 37460623 PMC10352345

[32] Tang C , Livingston MJ , Liu Z , Dong Z . Autophagy in kidney homeostasis and disease. Nat Rev Nephrol. 2020;16(9):489‐508. doi:10.1038/s41581-020-0309-2 32704047 PMC7868042

[33] Chakrabarti A , Oehme I , Witt O , et al. HDAC8: a multifaceted target for therapeutic interventions. Trends Pharmacol Sci. 2015;36(7):481‐492. doi:10.1016/j.tips.2015.04.013 26013035

[34] Dose A , Liokatis S , Theillet FX , Selenko P , Schwarzer D . NMR profiling of histone deacetylase and acetyl‐transferase activities in real time. ACS Chem Biol. 2011;6(5):419‐424. doi:10.1021/cb1003866 21302972

[35] Qi J , Singh S , Hua WK , et al. HDAC8 inhibition specifically targets Inv(16) acute myeloid leukemic stem cells by restoring p53 acetylation. Cell Stem Cell. 2015;17(5):597‐610. doi:10.1016/j.stem.2015.08.004 26387755 PMC4636961

[36] Zhang C , Guan Y , Zou J , Yang X , Bayliss G , Zhuang S . Histone methyltransferase MLL1 drives renal tubular cell apoptosis by p53‐dependent repression of E‐cadherin during cisplatin‐induced acute kidney injury. Cell Death Dis. 2022;13(9):770. doi:10.1038/s41419-022-05104-0 36068197 PMC9448773

[37] Lord CJ , Ashworth A . The DNA damage response and cancer therapy. Nature. 2012;481(7381):287‐294. doi:10.1038/nature10760 22258607

[38] Kim YJ , Kim TW , Park SR , Kim HT , Ryu SY , Jung JY . Expression of the Mre11‐Rad50‐Nbs1 complex in cisplatin nephrotoxicity. Environ Toxicol Pharmacol. 2015;40(1):12‐17. doi:10.1016/j.etap.2015.04.018 26056972

[39] Balasubramanian S , Ramos J , Luo W , Sirisawad M , Verner E , Buggy JJ . A novel histone deacetylase 8 (HDAC8)‐specific inhibitor PCI‐34051 induces apoptosis in T‐cell lymphomas. Leukemia. 2008;22(5):1026‐1034. doi:10.1038/leu.2008.9 18256683

[40] Kim JY , Han SY , Yoo J , et al. HDAC8‐selective inhibition by PCI‐34051 enhances the anticancer effects of ACY‐241 in ovarian cancer cells. Int J Mol Sci. 2022;23(15):8645. doi:10.3390/ijms23158645 35955780 PMC9369251

[41] Ko HJ , Chiou SJ , Tsai CY , et al. BMX, a specific HDAC8 inhibitor, with TMZ for advanced CRC therapy: a novel synergic effect to elicit p53‐, beta‐catenin‐ and MGMT‐dependent apoptotic cell death. Cell Commun Signal. 2022;20(1):200. doi:10.1186/s12964-022-01007-x 36575468 PMC9793577

[42] Song S , Wang Y , Xu P , et al. The inhibition of histone deacetylase 8 suppresses proliferation and inhibits apoptosis in gastric adenocarcinoma. Int J Oncol. 2015;47(5):1819‐1828. doi:10.3892/ijo.2015.3182 26412386

