# Supplementary material for: Inhibition of HDAC8 mitigates AKI by reducing DNA damage and promoting homologous recombination repair
Source: J Cell Mol Med. 2024 Sep 24;28(18):e70114. doi: 10.1111/jcmm.70114 (PMC11422176; doi:10.1111/jcmm.70114)
Supplement: Supplementary file 1 — Data S1. [file JCMM-28-e70114-s001.docx]

**Supplemental Figure 1.** **Immunohistochemistry of HDAC8 in the kidney.** Kidneys were collected at 48 h after cisplatin injection with or without PCI-34051. Photomicrographs illustrate imuunochemical staining of HDAC8 in mouse kidney sections. Scale bar = 100 μm.


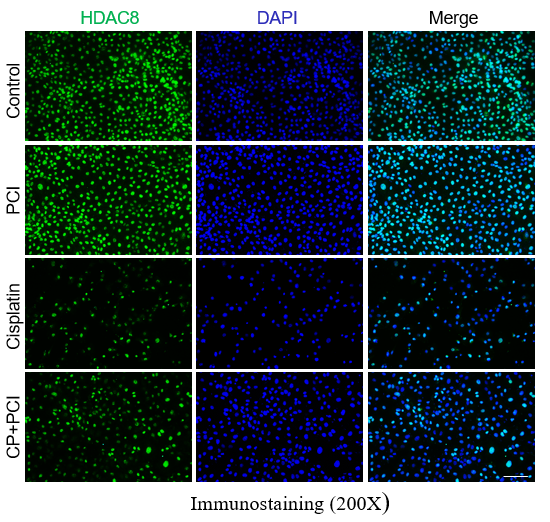


**Supplemental Figure 2.** **Immunostaining of HDAC8 in cultured renal epithelial cells.** Murine renal tubular epithelial cells (mRTECs) were treated according to the procedures outlined in the Materials and Methods section. Subsequently, they were subjected to immunostaining using an antibody specific to HDAC8, followed by counterstaining with 4',6-diamidino-2-phenylindole (DAPI), a nuclear dye. Scale bar = 100 μm.

润色

润色
